# Scaling statistical multiple sequence alignment to large datasets

**DOI:** 10.1186/s12864-016-3101-8

**Published:** 2016-11-11

**Authors:** Michael Nute, Tandy Warnow

**Affiliations:** 1 Department of Statistics, University of Illinois at Urbana-Champaign, 725 S. Wright St, Champaign, 61820 IL USA; 2 Department of Computer Science, University of Illinois at Urbana-Champaign, 201 North Goodwin Ave, Urbana, 61801 IL USA; 3 Department of Bioengineering, University of Illinois at Urbana-Champaign, 1270 Digital Computing Laboratory, MC-278, Urbana, 61801 IL USA; 4 National Center for Supercomputing Applications, University of Illinois at Urbana-Champaign, 1205 W. Clark St., MC-257, Urbana, 61801 IL USA; 5 Carl R. Woese Institute for Genomic Biology, University of Illinois at Urbana-Champaign, 1206 W. Gregory Dr., MC-195, Urbana, 61801 IL USA

**Keywords:** Multiple sequence alignment, Boosting, MCMC

## Abstract

**Background:**

Multiple sequence alignment is an important task in bioinformatics, and alignments of large datasets containing hundreds or thousands of sequences are increasingly of interest. While many alignment methods exist, the most accurate alignments are likely to be based on stochastic models where sequences evolve down a tree with substitutions, insertions, and deletions. While some methods have been developed to estimate alignments under these stochastic models, only the Bayesian method BAli-Phy has been able to run on even moderately large datasets, containing 100 or so sequences. A technique to extend BAli-Phy to enable alignments of thousands of sequences could potentially improve alignment and phylogenetic tree accuracy on large-scale data beyond the best-known methods today.

**Results:**

We use simulated data with up to 10,000 sequences representing a variety of model conditions, including some that are significantly divergent from the statistical models used in BAli-Phy and elsewhere. We give a method for incorporating BAli-Phy into PASTA and UPP, two strategies for enabling alignment methods to scale to large datasets, and give alignment and tree accuracy results measured against the ground truth from simulations. Comparable results are also given for other methods capable of aligning this many sequences.

**Conclusions:**

Extensions of BAli-Phy using PASTA and UPP produce significantly more accurate alignments and phylogenetic trees than the current leading methods.

**Electronic supplementary material:**

The online version of this article (doi:10.1186/s12864-016-3101-8) contains supplementary material, which is available to authorized users.

## Background

Multiple sequence alignment (MSA) of individual loci (where a locus is a recombination-free region within a genome) is the first step in many bioinformatics pipelines, including phylogeny estimation, protein classification, the detection of selection and co-evolution, and metagenomics. Several application areas could benefit directly from improved alignments and phylogenies of large-datasets. For example, metagenomic methods that rely on marker genes (e.g. [1–3]) invariably use genes that are present for well over 1,000 bacterial sequences and rely directly on the phylogeny to characterize the content of a shotgun sequencing sample. Improved alignments mean higher quality signal and thus more precise description of a given microbial community. Furthermore, it is well established that dense taxonomic sampling generally improves the estimation of phylogenies and multiple sequence alignments. Thus, multiple sequence alignment of datasets containing hundreds to many thousands of sequences is of increasing importance.

Numerous MSA methods have been developed, but only a few of these can analyze large datasets, and even fewer have been demonstrated to have good accuracy beyond a few hundred sequences [4]. The impact of multiple sequence alignment on downstream analyses is known to be substantial, with errors in multiple sequence alignment producing increased error rates in phylogeny estimation, false detection of positive selection, difficulties in detecting active sites in proteins, etc. [5]. Thus, highly accurate multiple sequence alignment, especially of large datasets spanning large evolutionary distances, is one of the major outstanding bioinformatics problems [6].

One of the most accurate approaches to multiple sequence alignment is statistical estimation under stochastic models of sequence evolution where sequences evolve down trees with insertions and deletions (jointly referred to as indels) and substitutions. Yet statistical estimation of alignments or trees under these models is rarely performed, largely because the current methods for this type of analysis are too computationally intensive to use on more than about 100 sequences. While many methods have this approach [7–10], BAli-Phy is the best-known, and the main such method that is used to estimate an alignment and phylogeny from unaligned sequences; [11] is the initial paper on this method, but subsequent publications extended and improved the statistical models on which the method is based.

Liu et al. [12] showed that BAli-Phy dominated SATé [12] and other alignment and tree estimation methods on datasets with 100 sequences with respect to alignment and tree accuracy, but the analysis took several weeks for each dataset. Even smaller datasets can be computationally intensive (for example, a BAli-Phy analysis of a dataset with 68 sequences took about 21 CPU days [13]), and the largest dataset that BAli-Phy has analyzed may be the 117-sequence dataset studied in [14]. However, BAli-Phy may not be able to run on substantially larger datasets than this; indeed, our initial testing found that with 500 sequences, BAli-Phy failed at an early step on every run (and practical constraints tend to limit its use below even that). Indeed, although BAli-Phy has been cited often in the literature, very few benchmarks of performance are included; most simply note that BAli-Phy has a strong statistical model but is slow and computationally demanding [15, 16]. Thus, improving the scalability of BAli-Phy to larger datasets is of great interest and potentially substantial impact.

Our group has developed several techniques [4, 12, 17, 18] to improve the scalability of multiple sequence alignment methods to large datasets, of which PASTA [18] and UPP [4] provide the largest improvements. PASTA is an iterative divide-and-conquer method for co-estimating trees and alignments, in which each iteration begins with a maximum likelihood tree computed in the previous iteration, and then uses the tree to partition the sequences into small subsets that are local within the tree. Then, a selected MSA method is applied to each subset and the subset alignments on adjacent subsets (defined by the topology in the tree) are aligned together using profile-profile alignment methods. Finally, an alignment on the entire dataset is obtained by transitivity. As shown in [18], using PASTA with MAFFT [19] on the subsets made it possible to align ultra-large datasets, including one with 1,000,000 sequences, and to do so with high accuracy. UPP uses a different approach: it selects a random subset of the sequences, computes an alignment and tree (called the backbone alignment and backbone tree) on the subset using PASTA, and then represents this PASTA alignment using an ensemble of Hidden Markov Models (HMMs), each computed on a small subset of the sequences (see [4] and “Methods” section for a description of how this ensemble is built). Each remaining sequence is then aligned to the backbone alignment using the best-scoring HMM in the ensemble. Finally, the entire set of sequences is aligned through transitivity. Like PASTA, UPP also produces highly accurate alignments of datasets with 1,000,000 sequences [4], and is more accurate than PASTA when the sequence dataset has fragmentary sequences [4].

In this study, we explore the use of both PASTA and UPP to boost BAli-Phy. PASTA is a method that has algorithmic parameters with default settings, and we use PASTA with its default settings as a starting tree. We then run one iteration of PASTA using BAli-Phy instead of MAFFT as the subset aligner, and we refer to this extension of PASTA by “PASTA+BAli-Phy”. As we will show, PASTA+BAli-Phy can align 1000-sequence datasets with higher accuracy than default PASTA. We also use PASTA+BAli-Phy (instead of default PASTA) to compute backbone alignments and trees with 1000 sequences within UPP, and we show that this approach produces more accurate alignments on 10,000-sequence datasets than default UPP. The improvements obtained over default PASTA and default UPP are significant, since these two methods are the current most accurate methods for large-scale and ultra-large scale multiple sequence alignment [4], especially (but not only) when alignments are used for phylogenetic estimation purposes.

The rest of this paper is organized as follows. In “Methods” section, we describe BAli-Phy, PASTA, and UPP, and the performance study we used to evaluate the impact of integrating BAli-Phy into PASTA and UPP. In “Results and discussion” section, we report the results of the performance study. In “General observations” section, we discuss the implications of the study and future research. Additional results and discussion are provided in Additional file 1.

## Methods

We ran two experiments in this study. The first experiment evaluated PASTA+BAli-Phy on 1000-sequence datasets in comparison to other alignment methods, and the second experiment evaluated UPP using PASTA+BAli-Phy to compute the backbone alignment and tree in comparison to other alignment methods on 10,000-sequence datasets. All datasets are available from prior publications.

### Algorithms

**BAli-Phy** is a method that uses Gibbs sampling to alternately sample a new alignment, followed by a new phylogeny, each proportional to their likelihood under its sequence evolution model. Unlike standard phylogenetic models, such as the Generalized Time Reversible (GTR) model [20] in which only substitutions occur, the stochastic models in BAli-Phy, RS05 and subsequently RS07, also have indels. The resulting set of simulated phylogeny-alignment pairs constitutes an estimate of the joint posterior distribution. BAli-Phy does not have a well-defined stopping rule, and will run indefinitely until it is terminated. Hence, to compute a single MSA using BAli-Phy, it is necessary to define a stopping rule and a method for extracting the final alignment. In the study presented here, BAli-Phy was stopped after 24 hours of running independently on all 32 cores of a single node on the Blue Waters computing facility at UIUC [21]. Once completed, the posterior decoding (PD) alignment was computed using the alignment-max command within BAli-Phy and designated as the output alignment. The PD alignment is obtained by scoring each column in the sample alignments according to how often it appears, and choosing the set of columns that a) constitutes a valid MSA on the data and b) has the largest cumulative score possible. We chose the PD alignment because prior studies have shown that the PD alignment was more accurate than the MAP (maximum *a posteriori*) alignment [12, 22].

For all experiments described in this paper, we use “BAli-Phy" to specifically refer to the protocol described above for computing a multiple sequence alignment from a given input, using BAli-Phy v2.3.6. No restrictions or starting data were provided to the software; commands for its execution, as well as for computation of the PD alignment, are provided in Additional file 1.

**MAFFT** is a well known method for multiple sequence alignment that has been consistently one of the top performing methods in terms of alignment accuracy on both nucleotide and amino acid benchmarks [12, 23]. MAFFT has many ways of being run, but its most accurate settings, such as using the local pairs (MAFFT L-INS-i) command, are computationally very intensive on large datsets. MAFFT run in default mode will select the variant to run based on the dataset size, but will not typically have the same high accuracy as when run using the local pairs command.

**PASTA** is an algorithm for large-scale multiple sequence alignment that has several algorithmic parameters that can be set by the user, but also has default settings, which we now describe. PASTA operates by initializing an alignment, then iteratively estimating a maximum likelihood (ML) tree using FastTree-2 [24] on the alignment, estimating an alignment with the help of this tree, and repeating. The calculation of the new alignment given the current tree is obtained using a specific divide-and-conquer strategy, wherein the tree is broken into subtrees through repeatedly deleting centroid edges until each subtree has a small enough number of sequences (the default maximum size is 200). Then, the preferred multiple sequence alignment method (default is MAFFT L-INS-i) is used to align each subset, yielding a set of subset MSAs. Then, every pair of subset alignments that are adjacent to each other in the tree are merged into a larger alignment using a profile-profile alignment technique (default is OPAL [25]). This produces a set of larger subset alignments that overlap and agree pairwise in all homologies for those sequences that they share and enables an alignment on the entire set to be computed using transitivity. The number of times this process iterates can be set by the user, but the default is three. As shown in [18], PASTA improves on both SATé [12] and SATé-II [17] in terms of accuracy and scalability to large datasets.

**PASTA variants:** PASTA has default settings as described above that were selected for use with MAFFT L-INS-i as the subset aligner. However, PASTA can be used with any MSA method as the subset aligner. In this paper, we examine the effect of using BAli-Phy instead of MAFFT L-INS-i within PASTA. In order to implement this, some additions to the infrastructure within PASTA were necessary. See Additional file 1 for details.

Because BAli-Phy requires 24 hours and a 32-core server to run whereas MAFFT L-INS-i runs on 200 sequences in a matter of minutes, replacing MAFFT L-INS-i for the initial iterations when the subsets are effectively (more) random would have been a poor use of expensive computing resources. We therefore chose to implement it by running PASTA in default mode (which involves three iterations), and then performing the fourth iteration using BAli-Phy as the subset aligner. Because BAli-Phy is able to run on datasets with 100 sequences, we set the decomposition size to 100 instead of 200, which is the default setting. All other parameters were run in default mode. The two natural lines of inquiry with the tests were therefore (a) does the fourth iteration using BAli-Phy improve the alignment compared with the result after the first three iterations (i.e., PASTA in its default settings), and if so, (b) can we be sure it is due to BAli-Phy and not simply that we used an extra iteration? To explore these questions, we tested the following three variants of PASTA:

1. **PASTA(default):** PASTA with fully default settings, which means three iterations, maximum subset size 200, with MAFFT L-INS-i as the subset-aligner, and OPAL to align pairs of subset alignments. We denote this by **P(default)**.
2. **PASTA+BAli-Phy:** PASTA with three iterations under default settings, followed by one iteration with maximum subset size 100 and BAli-Phy as the subset aligner. (Equivalently, the final iteration was simply run with the phylogeny estimated in (1) specified as an input.) We denote this by **P+BAli-Phy**.
3. **PASTA+MAFFT-L:** PASTA with three iterations under default settings, followed by one iteration with maximum subset size 100 and MAFFT L-INS-i as the subset aligner. (Also equivalently specified as a single-iteration.) We denote this by **P+MAFFT-L**.

**UPP** is a fast multiple sequence alignment method that can be extended to 1,000,000 sequences easily, and is especially robust to fragmentary sequences compared to PASTA [4]. UPP works by choosing a random subset of (at most) 1000 sequences in the dataset to be the “backbone" and aligns those sequences with PASTA. It then constructs a collection of HMMs (called an “ensemble of HMMs") on the backbone alignment. For each of the remaining sequences, it finds the HMM from the ensemble that has the best bitscore, and uses that HMM to add the sequence to the backbone alignment. These additions are done independently, because the backbone alignment does not change during the process. UPP runs in time that is linear in the number of sequences in the input, and is also highly parallelizable. We present results using UPP with the three variants of PASTA described above to compute the backbone alignment and tree on 1000-sequence subsets of different 10,000-sequence datasets.

**Maximum likelihood trees** were estimated on each estimated and true 1000-sequence alignment using RAxML [26] and FastTree-2 [24], two of the most accurate methods for large-scale maximum likelihood [27]. For the 10,000-sequence datasets, we only used FastTree-2, since RAxML is too slow on such datasets. We ran RAxML and FastTree-2 in their default modes under the GTR model with gamma-distributed rates across sites.

### Data

In order to test the algorithms described above, a collection of simulated datasets used in [18] was downloaded from the authors’ website. This collection included data generated by three separate sequence evolution simulators, Indelible [28], RNASim [29], and RoseDNA [30]. Each of these simulators has distinct properties, and hence represents a unique set of simulation conditions. Two of the three (Indelible and RNAsim) included 10,000 sequences in each replicate, while the third (RoseDNA) included only 1,000. For the former, ten replicates from each simulator were used and a single set of 1,000 sequences was randomly chosen from the original.

Table 1 contains some descriptive statistics for the reference alignments of each of the 1,000-sequence simulated data. The RNAsim data are considerably different from the other two, with longer sequences and shorter evolutionary diameter, as well as many more indels of shorter length. The RoseDNA and Indelible data, on the other hand, are similar to each other, with the primary difference being the overall rate of evolution. Finally the individual RoseDNA model conditions vary chiefly with respect to the length of the indels. In all, each of the three simulators provides insight into a unique part of the data space. Detailed descriptions of the simulators and the data used are provided below.

**Table 1:** Summary statistics for true alignments on 1,000-sequence data

		*p-distance*		Gaps/Seq	Gap Length	% Blank	
	Sites	Avg	Max				
RNAsim	4806	41 %	61 %	1036	3.1	68 %	
Indel. M2	2179	67 %	74 %	210	5.6	54 %	
Rose L1	3777	70 %	77 %	209	13.2	73 %	
Rose M1	3934	70 %	77 %	294	9.9	74 %	
Rose S1	2106	69 %	77 %	285	3.9	52 %	

The p-distance is the normalized pairwise Hamming distance. Numbers shown are averages over 10 replicates

**RoseDNA** is a subset of a larger collection of DNA sequences simulated using the ROSE simulator [30] that was used in [12] to evaluate SATé in comparison to other MSA methods. The ROSE simulator is a straightforward implementation of the HKY stochastic model, which is itself a close precursor to the standard Generalized Time Reversible (GTR) model [20] in use today. The simulator adds an additional model that allows the user to simulate insertions (and similarly deletions) by simulating, in order, the number of insertion events that occur, the position of each insertion followed by its length. We used 10 replicates of the 1,000-sequence datasets from the model conditions labeled 1000M1, 1000S1 and 1000L1 from [12], where the M/S/L moniker refers to the average gap length (i.e. medium, short or long, respectively) of each indel event. The specific model conditions we selected have high rates of evolution, and were selected to provide a substantial challenge to the MSA methods.

**Indelible** is similar to ROSE, but includes some additions that accommodate additional model complexity, such as gamma-distributed rates across sites and a codon model. The Indelible data used for these experiments are the same data used in [18], and includes only the model condition labeled M2 in the previous paper, which is the highest rate of evolution of the three that were used.

**RNAsim** simulates RNA sequence evolution down a tree, specifically taking RNA structure into account, and hence represents a significant departure from the previous two. It uses a population genetics model with selection to simulate sequence mutations, with selection favoring mutations with a relatively low free energy in its folded state. This is designed to emulate actual conditions that might plausibly be acting on mutations to RNA sequences, particularly those in a folded state such as ribosomal RNA. As a result, it has several major differences from the other simulators. First, there is no uniform substitution matrix used in the simulation. Second, site mutation probabilities are not independent of one another. Importantly, by contrast with the other two simulators, these differences are a departure from the likelihood model (GTRGAMMA) used in the maximum likelihood phylogeny estimation step of PASTA, and also a departure from the substitution model used by BAli-Phy. Therefore, results on the RNAsim data provide a test of the MSA method’s robustness to model misspecification, and indirectly also test the ability of GTRGAMMA maximum likelihood phylogeny estimation to be robust to substantial model misspecification.

#### Evaluation criteria

We explore alignment accuracy using three standard criteria: modeller score (i.e., precision), SP score (i.e., recall), and total column (TC) score, as computed by FastSP [31]. The modeller score is equivalent to 1-SPFP, where SPFP is the “sum-of-pairs false positive rate"; similarly, the SP score is equivalent to 1-SPFN, where SPFN is the “sum-of-pairs false negative rate". These SPFP and SPFN error rates are based on homologies between nucleotides that appear in the true and estimated alignments [31]. The TC score is the fraction of the number of columns in the true alignment that are recovered in the estimated alignment. All accuracy criteria are given as a percentage, with 100 % indicating perfect accuracy.

We explore phylogenetic accuracy of maximum likelihood (ML) trees computed on these alignments using the Robinson-Foulds (RF) error rate, where the RF error is the percentage of the non-trivial bipartitions in the true tree that are missing from the estimated tree. We report accuracy using “Delta-RF", which is the change in the RF error rate between the ML tree computed using the estimated alignment and the ML tree computed on the true alignment. The RF error rates were calculated using DendroPy [32].

## Results and discussion

**Results for experiment 1:** We compare P+BAli-Phy, P+MAFFT-L, P(default), MAFFT L-INS-i, and MAFFT run in default mode; see Table 2. P+BAli-Phy has the top TC scores of all methods, with very substantial improvements over the second best method, which is typically P+MAFFT-L. P+BAli-Phy is also the best performing method in terms of alignment precision and recall on four of the five model conditions, and in second place on the fifth (Rose S1), where P(default) is best. However, P+BAli-Phy is within 1 % of P(default) on the Rose S1 datasets in terms of precision and recall. MAFFT L-INS-i produces less accurate alignments than the PASTA variants we study, but is *much* more accurate than MAFFT run in default mode. The fact that default MAFFT has poor accuracy on these datasets shows that these are not datasets that are aligned with high accuracy by all methods; only the better methods provide good accuracy on these datasets.

**Table 2:** Alignment and tree accuracy metrics for all methods on 1,000 sequences

					Delta-*RF*	
Data	Method	Prec.	Rec.	TC	RAxML	FT-2
Indelible	P(Default)	95.1 %	94.6 %	4.5 %	1.86 %	0.68 %
M2	P+BAli-Phy	**98.7 %**	**98.7 %**	**14.6 %**	**0.29 %**	**-0.72 %**
	P+MAFFT-L	97.2 %	97.0 %	6.8 %	0.75 %	-0.20 %
	MAFFT-L	80.2 %	75.0 %	1.4 %	15.73 %	8.74 %
	MAFFT-def	1.0 %	0.4 %	0.0 %	(not run)	(not run)
RNAsim	P(default)	90.3 %	90.4 %	3.5 %	0.56 %	**0.33 %**
	P+BAli-Phy	**92.1 %**	**92.1 %**	**8.5 %**	0.70 %	0.42 %
	P+MAFFT-L	88.8 %	89.0 %	3.9 %	**0.34 %**	0.45 %
	MAFFT-L	91.8 %	91.5 %	2.9 %	0.73 %	6.47 %
	MAFFT-def	83.7 %	71.5 %	1.4 %	(not run)	(not run)
Rose L1	P(default)	90.9 %	90.6 %	15.9 %	2.07 %	2.24 %
	P+BAli-Phy	**91.8 %**	**91.7 %**	**33.2 %**	**1.47 %**	**1.51 %**
	P+MAFFT-L	90.0 %	89.8 %	21.8 %	1.98 %	2.00 %
	MAFFT-L	84.1 %	76.6 %	6.4 %	3.45 %	3.15 %
	MAFFT-def	1.1 %	0.4 %	0.0 %	(not run)	(not run)
Rose M1	P(default)	79.7 %	79.0 %	9.0 %	5.35 %	6.26 %
	P+BAli-Phy	**79.8 %**	**79.6 %**	**24.4 %**	4.70 %	5.45 %
	P+MAFFT-L	78.6 %	78.2 %	12.9 %	5.96 %	5.89 %
	MAFFT-L	74.9 %	63.3 %	3.0 %	**3.64 %**	**3.90 %**
	MAFFT-def	1.2 %	0.5 %	0.0 %	(not run)	(not run)
Rose S1	P(default)	**85.3 %**	**85.1 %**	2.8 %	3.94 %	4.29 %
	P+BAli-Phy	84.3 %	84.3 %	**10.3 %**	**2.26 %**	**3.59 %**
	P+MAFFT-L	83.5 %	83.3 %	4.8 %	3.55 %	4.38 %
	MAFFT-L	76.2 %	68.2 %	0.5 %	3.80 %	3.79 %
	MAFFT-def	1.2 %	0.5 %	0.0 %	(not run)	(not run)

Note that precision, recall and TC are accuracy metrics (so larger is better) but Delta-RF is an error metric (so smaller is better). Metrics are averages over 10 replicates. Method names have been shortened slightly for space: P(default) refers to PASTA(default), P+(...) is shorthand for PASTA+(...), MAFFT-def refers to default MAFFT, and MAFFT-L refers to MAFFT L-INS-i. Bold numbers indicate best performing method

Results in terms of tree error are somewhat more mixed: P+BAli-Phy is best on three of the five model conditions, in second place (behind MAFFT L-INS-i) on one condition (Rose M1), and in second or third place (depending on which ML software is used) on the remaining condition (RNASim). However, on those conditions where P+BAli-Phy does not have the highest tree accuracy, it is close to the best performing method (within 0.36 % in terms of Delta-RF on the RNASim data, and within 1.6 % on the Rose M1 data).

Overall, default MAFFT has the worst accuracy of all methods on these data with respect to all criteria. MAFFT L-INS-i is clearly more accurate than default MAFFT, but not as accurate as the PASTA variants in terms of alignment criteria. Hosever, MAFFT L-INS-i has the best tree accuracy on the Rose M1 datasets, and second best tree accuracy on the Rose S1 datasets.

**Fig. 1:**
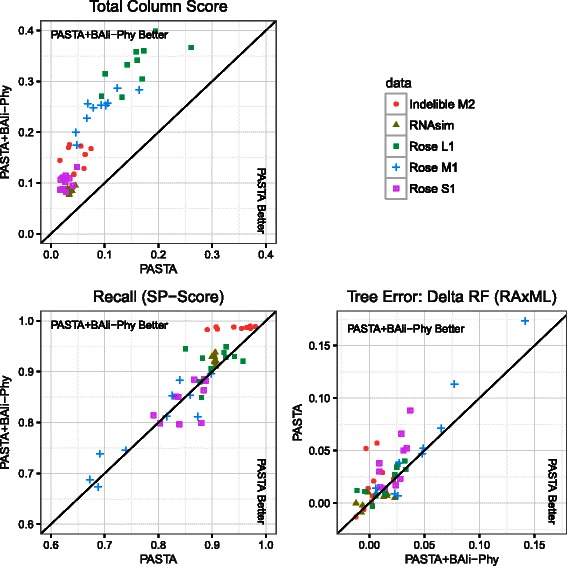
Results on 1000-sequence datasets, comparing default PASTA and PASTA+BAliPhy. Each point represents one replicate. PASTA denotes the alignment from PASTA under default settings (referred to as “PASTA(default)” in the text), and PASTA+BAli-Phy denotes the alignment after an additional iteration using BAli-Phy. Delta-RF refers to the difference between the RF error rates of ML trees computed on the estimated and true alignments. In each subfigure, a position above the 45-degree line indicates that PASTA+BAli-Phy is preferable; the axes for the subfigure for Delta-RF have been flipped to maintain this interpretation, since Delta-RF is an error metric rather than an accuracy metric

Figure 1 shows results for each replicate comparing P+BAli-Phy to P(default), with respect to three metrics: TC score, Delta-RF, and SP-score. Results for Modeler score are nearly identical to SP-score, and are shown in Additional file 1. Results for FastTree-2 and RAxML as the ML tree estimation method are similar; here we show results for RAxML; see Additional file 1 for FastTree-2. Points above the *x*=*y* diagonal correspond to datasets in which P+BAli-Phy is more accurate than P(default) for the specified criterion, and conversely points below the diagonal correspond to datasets in which P+BAli-Phy is less accurate. Note that P+BAli-Phy has a higher TC score on *every* replicate than P(default) (all points are above the *x*=*y* diagonal), and the improvement in TC score is particularly substantial (the distance to the *x*=*y* diagonal is large). P+BAli-Phy also produces more accurate trees on nearly all replicates of all model conditions (note the particularly large improvements on several of the Rose S1 replicates). With the exception of the Rose S1 model condition, P+BAli-Phy is as good or better than default PASTA in terms of SP-score (more replicates above the *x*=*y* diagonal than below). Furthermore, although default PASTA has slightly better SP-scores than P+BAli-Phy on several of the Rose S1 replicates, P+BAli-Phy is nearly always better with respect to tree accuracy on these replicates.

The same figure comparing PASTA with BAli-Phy to PASTA with MAFFT shows virtually identical patterns and is contained in Additional file 1.

**Fig. 2:**
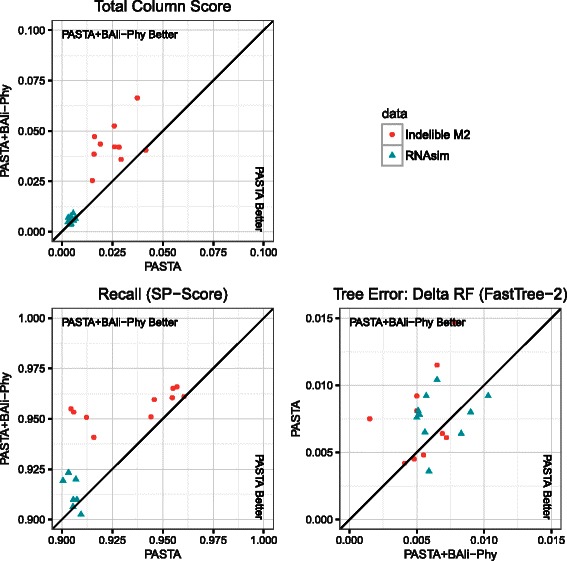
Results on 10,000 sequences. Using UPP on two different backbones: one computed using default PASTA and the other computed using PASTA+BAliPhy (i.e., one iteration of PASTA using BAli-Phy as the subset aligner after default PASTA completes). Each point represents one replicate. Delta-RF refers to the difference between the RF error rates of ML trees computed on the estimated and true alignments. In each subfigure, a position above the 45-degree line indicates that PASTA+BAli-Phy is preferable; the axes for the subfigure for Delta-RF have been flipped to maintain this interpretation, since Delta-RF is an error metric rather than an accuracy metric

**Results for experiment 2:** In Experiment 2, we compared three variants of UPP that differ only in how the backbone alignment and tree are computed; see Table 3. Clearly using P+BAli-Phy to compute the backbone alignment and tree has the highest alignment accuracy for all three criteria, and the gains in accuracy are largest in terms of the TC score; the second most accurate method uses P+MAFFT-L to compute the backbone alignment and tree. UPP only computes an alignment, so we computed ML trees on these three alignments using FastTree-2 (RAxML is too slow to run on 10,000 sequences). Note that the trees computed using UPP with P+BAli-Phy are within 0.67 % in RF error of the tree computed using ML on the true alignment, showing that the alignment error is low enough to not impact the tree estimation by much in comparison to the tree computed on the true alignment. Figure 2 shows results for each replicate and demonstrates that improvements in alignment accuracy occur on nearly every replicate.

**Table 3:** Alignment and tree accuracy metrics for UPP alignments on 10,000 sequences

Data	Backbone	Prec.	Rec.	TC	*Δ*-RF
	P(default)	96.2 %	93.6 %	2.6 %	0.77 %
Indelible	P+BAli-Phy	**97.8 %**	**95.6 %**	**4.3 %**	**0.54 %**
	P+MAFFT-L	97.3 %	95.0 %	3.2 %	0.62 %
	P(default)	90.8 %	90.5 %	0.5 %	0.77 %
RNAsim	P+BAli-Phy	**91.4 %**	**91.0 %**	**0.6 %**	**0.67 %**
	P+MAFFT-L	89.4 %	89.1 %	0.5 %	**0.67 %**

Each method shown under Backbone is the method used to align the backbone of 1,000 sequences. Due to the running time required for RAxML on data of this size, *Δ*-RF shown is for FastTree-2 only. Bold numbers indicate best performing method

### Statistical significance

Table 4 shows *p*-values for each metric and each model condition for the hypothesis that P+BAli-Phy outperforms P(default) on measures of alignment accuracy, correcting for multiple tests using the Benjamini-Hochberg procedure [33], and Table 5 shows the same for measures of tree accuracy. P+BAli-Phy has statistically significant improvements over P(default) with respect to the TC score on all the model conditions. P+BAli-Phy also has statistically significant improvements over P(default) with respect to precision and recall (alignment modeller and SP-score) on the Indelible and RNASim datasets, but not on the RoseDNA datasets. ML trees computed on P+BAli-Phy alignments are also statistically significantly more accurate than ML trees computed on P(default) alignments for 6 of the 10 combinations of model condition and ML software.

**Table 4:** *P*-values for each model condition and metric for the hypothesis test that P+BAli-Phy outperforms P(default) with respect to *alignment* accuracy

Data	Precision	Recall	TC		
Indelible M2	**0.001**	**0.001**	**<0.001**		
RNAsim	**<0.001**	**0.001**	**<0.001**		
Rose L1	0.211	0.188	**<0.001**		
Rose M1	0.473	0.298	**<0.001**		
Rose S1	0.820	0.770	**<0.001**		

Values are based on one-sided Student’s T-test for differences between the two methods on each replicate. Bolded values indicate significant differences using a Benjamini-Hochberg procedure to control the false discovery rate at 5 % [33]

**Table 5:** *P*-values for each model condition and metric for the hypothesis test that P+BAli-Phy outperforms P(default) with respect to *tree* accuracy

	Delta-*RF*	Delta-*RF*			
Data	RAxML	FastTree-2			
Indelible M2	**0.021**	**0.014**			
RNAsim	0.677	0.660			
Rose L1	0.036	0.054			
Rose M1	0.136	**0.030**			
Rose S1	**0.010**	**0.007**			

Values are based on one-sided Student’s T-test for differences between the two methods on each replicate. Bolded values indicate significant differences using a Benjamini-Hochberg procedure to control the false discovery rate at 5 % [33]

### General observations

As this study showed, incorporating BAli-Phy into PASTA produced alignments that were generally more accurate than default PASTA, which is based on MAFFT; similarly, incorporating PASTA+BAli-Phy into UPP produced alignments that were more accurate than default UPP, which is based on default PASTA. The improvement in alignment accuracy was most noticeable for the Total Column (TC) score, where PASTA+BAli-Phy had much higher TC scores than the next best method, which was PASTA+MAFFT-L. For example, on the 1,000-sequence datasets we studied, PASTA+BAli-Phy had much higher TC scores than PASTA+MAFFT-L and default PASTA, by factors that ranged from 1.5 to 2.2 (for PASTA+MAFFT-L) and from 2.1 to 3.7 (for default PASTA). PASTA+BAli-Phy nearly always produced alignments that have higher modeller-score (precision) and SP-score (recall), with the single exception being the RoseDNA S1 dataset with 1,000 sequences, where it was 1 % lower than the best-performing (default PASTA), but both had good accuracy (precision and recall greater than 84 %). The integration of PASTA+BAli-Phy into UPP produces alignments that strictly dominate the second best performing method, which is UPP run in default mode, using default PASTA to compute its backbone tree. Thus, integrating BAli-Phy into PASTA and UPP improves alignment accuracy with respect to all three criteria, with particularly large improvements for TC scores. Perhaps the most important trend with respect to tree accuracy is that for all 10,000-sequence model conditions and nearly all 1,000-sequence model conditions, ML trees computed on the PASTA+BAli-Phy alignments are within 1 % (in terms of tree error) of the ML tree computed on the true alignment. Thus, in general, alignment error in PASTA+BAli-Phy does not increase tree error in a noticeable way over what could be computed given the true alignment. The only exceptions to this are the RoseDNA datasets, where the increase in tree error obtained on the PASTA+BAli-Phy alignment compared to trees computed on the true alignment ranges from 1.47 % (RoseDNA L1) to 4.7 % (RoseDNA M1). However, ML trees on other alignments on those datasets also have somewhat higher Delta-RF error on these RoseDNA datasets. Indeed, PASTA+BAli-Phy has the lowest Delta-RF error on four of the six combinations of ML method and model condition, and comes in second place on the remaining two conditions. Furthermore, when ML trees computed on PASTA+BAli-Phy alignments are not the most accurate, they are very close in accuracy to the the most accurate trees, with differences that range from 0.36 to 1.6 %.

The gap length distribution affects alignment difficulty, with short gap datasets harder to align correctly than datasets with long gaps. The comparison between results on the 1,000-sequence RoseDNA M1 (medium gap length) datasets and the 1000-sequence RoseDNA S1 datasets is interesting, though. If alignment precision and recall are considered, then the RoseDNA M1 datasets are more difficult, as they result in reduced precision and recall values for all methods; however, if TC scores are considered, then the RoseDNA S1 datasets are more difficult. Clearly, model conditions impact performance with respect to the different alignment criteria differently, but generally short gaps combined with high rates of substitution create the hardest conditions.

## Conclusions

This study was limited to simulated datasets where sequences evolve down model trees under processes that include insertions, deletions, and substitutions. Of the three simulators used to produce these datasets, RNASim is the most complex, and in particular includes sites that co-evolve based on the secondary structure for the RNA molecule used to design the simulation. On these datasets, we explored the use of BAli-Phy within PASTA (and then within UPP) as a point estimator of the true sequence alignment. Our study shows that incorporating BAli-Phy into PASTA and UPP enables BAli-Phy to be extended to large and ultra-large datasets, and to produce more accurate alignments than the default settings for PASTA and UPP, which are the current best alignment methods for large-scale and ultra-large-scale multiple sequence alignment. Indeed, what this study shows is that integrating BAli-Phy into PASTA means that a dataset with 1000 sequences can be aligned in about the same time as 10 independent BAli-Phy analyses of 100 sequences each. Furthermore, once a dataset of this size is computed, larger datasets can be aligned very quickly by using the PASTA+BAli-Phy alignment as the backbone alignment and tree in UPP. Thus, even though this approach does not address how to speed up BAli-Phy for 100-taxon datasets, it does show that BAli-Phy can be scaled to much larger datasets in an essentially linear fashion.

There are several limitations to this study. First, although we explored this technique with BAli-Phy, we did not explore it with other statistical methods. However, since the parameters of the divide-and-conquer strategy (especially the maximum subset size) can be adjusted to suit the given base MSA method, this extension can be easily done. Thus, methods such as StatAlign [8], which may be limited to even smaller datasets, could also be tested in this framework. Similarly, methods such as PAGAN [34] are impacted by dataset size and the challenge in estimating good guide trees, and PASTA’s phylogenetically-informed divide-and-conquer strategy might be useful techniques to improve their scalability to large sequence datasets, especially when the sequence datasets are highly heterogeneous. Thus, future work should evaluate the impact of this type of strategy on StatAlign, PAGAN, and other statistical methods.

Our study also only examined minor adjustments to the algorithmic parameters for PASTA and UPP; additional research to optimize the parameters involved in this implementation could lead to substantial improvements, as essentially no parameter tuning was done.

This study was limited to simulated datasets, and so the potential for this type of approach to provide improvements on biological datasets is unknown. One of the challenges is that most biological alignment benchmarks are amino acid datasets; while BAli-Phy can analyze amino acid sequences, it is even more computationally intensive on amino acid datasets than on nucleotide datasets, and it is not known whether the statistical approach in BAli-Phy will provide advantages for structural alignment estimation.

Finally, one of the appealing aspects of the Bayesian approach in BAli-Phy is that it returns a sample from the distribution on multiple sequence alignments and trees. This study only explored BAli-Phy as a point estimator of the alignment, and so in a sense does not truly scale BAli-Phy to large datasets. Scaling Bayesian methods such as BAli-Phy so that they achieve their full potential on large datasets is clearly of great interest, and future work should attempt to do this.

## Additional file

Additional file 1 Supplementary information. Additional file 1 contains the details of the modifications to PASTA discussed in Section “Methods” as well as specific commands used to run each software used in the paper. It also contains additional data, including additional pairwise plots similar to Fig. 1 and a performance comparison of the two maximum-likelihood tree estimation programs.
