# Supplementary material for: Scaling statistical multiple sequence alignment to large datasets
Source: BMC Genomics. 2016 Nov 11;17(Suppl 10):764. doi: 10.1186/s12864-016-3101-8 (PMC5123300; doi:10.1186/s12864-016-3101-8)
Supplement: Supplementary file 1 — Supplementary information. Additional file 1 contains the details of the modifications to PASTA discussed in Section “Methods” as well as specific commands used to run each software used in the paper. It also contains additional data, including additional pairwise plots similar to Fig. 1 and a performance comparison of the two maximum-likelihood tree estimation programs. [file 12864_2016_3101_MOESM1_ESM.pdf]

Scaling Statistical Multiple Sequence Alignment to Large Datasets

Additional File 1

Michael Nute[1], Tandy Warnow[2,3]

September 21, 2016

[1]Department of Computer Science, University of Illinois at Urbana-Champaign
[2]Department of Statistics, University of Illinois at Urbana-Champaign
[3]Department of Bioengineering, University of Illinois at Urbana-Champaign

Contents

1 Technical Modifications to PASTA **1**

2 Software Commands Used **1**

3 Additional Scatter Plots for 1000-sequence Data **3**

4 Comparison of RAxML vs. FastTree-2 **4**

1 Technical Modifications to PASTA

The default version of PASTA v1.6.3 is designed to run on a single node using a shared-memory implementation with multiple processors, and to run consecutively from start to finish using a single command. However, running PASTA in this manner is not practical using BAli-Phy as the subset aligner; with 1,000 sequences and a maximum subset size of 100, PASTA requires 10-16 subset alignments that require 32 CPU-days apiece. While this is theoretically possible given the right computing resource, shared resources such as university clusters frequently have constraints on job time, to name one, that prohibit a continuous implementation of PASTA. Instead, we needed an implementation of PASTA that allows subsets to be aligned separately, on different servers or at different times if necessary.

The solution was to add an optional checkpoint just prior to the subset alignment step where it will save its state, terminate, and when restarted, resume from the previous point assuming that the subsets had been aligned and copied to the appropriate path. When PASTA is run with this option, upon reaching the checkpoint it outputs a text file with paths to the subset sequence files and target paths for the aligned version. This addition is therefore useful not just for using BAli-Phy as the subset aligner, but any method that is not currently implemented, or even to achieve additional parallelism on data where the number of subsets makes a single node impractical. The commands and links to source code for this branch of the PASTA code are provided in the following section.

2 Software Commands Used

In the sections below, `<datatype>` refers to whether the sequences are DNA, RNA or amino acid sequences. This was DNA for all of our data except for RNAsim, which was RNA.

PASTA

The following commands assume that the work for PASTA(default), PASTA+MAFFT-L and PASTA+BAli-Phy sit in folders called respectively `<pasta_def_folder>`, `<pasta_mafft_folder>` and `<pasta_bp_folder>`. For PASTA(default) and PASTA+MAFFT-L, the commands are:

Default:

```
python run_pasta.py -d <datatype> -i <raw_seqs> -j pasta_default -o
<pasta_def_folder>/<model-replicate folder> --temporaries=<temps_folder> -k
```

MAFFT:

```
python run_pasta.py -i <raw_seqs>
-t <pasta_def_folder>/<model replicate folder>/pasta_default.tre
-o <pasta_mafft_folder>/<model/replicate folder> -j pj_mafft -d <datatype>
```

```
--temporaries=<temps_folder> --max-subproblem-size=100 -k --keepalignmenttemps
--iter-limit=1
```

For PASTA+BAli-Phy, there are two commands. The first starts PASTA and does the decomposition, and then exits after outputting a list of files that need to be aligned and saving its state. The second resumes from the previously saved state under the assumption that the alignments have been completed and are in their target locations on disk and proceeds with the remainder of the algorithm. The commands are:

Start:

```
python run_pasta.py -i <raw_seqs>
-t <pasta_def_folder>/<model replicate folder>/pasta_default.tre -o
<pasta_bp_folder>/<model-replicate folder> -j pastabp -d <datatype> --interruptible
--temporaries=<temps_folder> --max-subproblem-size=100 -k --keepalignmenttemps
--iter-limit=1
```

Finish:

```
python run_pasta.py -i <raw_seqs> -o <pasta_bp_folder>/<model replicate folder> -j pastabp
-d <datatype> -t <pasta_def_folder>/<model replicate folder>_default.tre --interruptible
--temporaries=<temps_folder> --max-subproblem-size=100 --resume-state-path=<pasta_bp_folder>/
<model replicate folder>/pastabp_temp_iteration_0_picklefile -k --keepalignmenttemps
--iter-limit=1
```

The option `-interruptible` is necessary in both and indicates to PASTA that it should stop at the checkpoint. In the second command, the option `-resume-state-path` is the path to the file containing the state at checkpointing and the sample argument there is consistent with the naming that PASTA will use for that file.

BAli-Phy

BAli-Phy was run on Blue Waters by setting up a job with a wall-clock time limit of 24 hours and submitting it with the following shell script, which starts a background BAli-Phy process once for every processor found by the `nproc` Linux command (on Blue Waters, it is 32). In the following, the variable `${id}` is assumed to be a name that identifies the particular alignment being run.

```
for iteration in $(seq 1 $(nproc))
do
bali-phy <subset-fasta> --name <job_id_work_folder>/${id}_out/${id}-${iteration} >
<job_id_work_folder>/${id}_out/log_$iteration.txt 2>&1 &
done
wait
```

After the job has completed the following is run, which removes the first 10 samples from the output for each processor, then concatenates the rest into a single file. Then the posterior-decoding alignment is computed in the final line with the `alignment-max` command.

```
for iteration in $(seq 1 32)
do
cut-range --skip=10 < <job_id_work_folder>/${id}_out/${id}-${iteration}-1/C1.P1.fastas
>> <job_id_work_folder>/${id}_out/combined.fasta
done
cat <job_id_work_folder>/${id}_out/combined.fasta | alignment-max >
<job_id_work_folder>/${id}_out/${id}_posterior_decoding.fasta
```

MAFFT

We ran MAFFT in two ways: its default version, and its MAFFT L-INS-i version. The command for the L-INS-i algorithm is:

```
mafft-linsi <raw_seqs> > <output_file>
```

UPP

Other that specifying the backbone alignment and tree, UPP was run with default settings. The command is:

```
python run_upp.py -m <datatype> -s <raw_query_seqs> -d <work_folder> -t <tree>
-a <backbone_alignment> -p <temps_folder>
```

In this command, <raw_query_seqs> is a fasta file with the unaligned sequences, *excluding* the sequences included in the backbone alignment. <tree> refers to a specified phylogenetic tree on the backbone alignment and <backbone_alignment is the corresponding alignment itself. Both were the default output by PASTA for that particular backbone. By default, PASTA uses FastTree-2 to find its final tree estimate using the parameters -nt -gtr -gamma -fastest, and this was the tree used in the command above.

RAxML & FastTree-2

Finally, for maximum-likelihood trees estimated directly (i.e. not from within PASTA), both methods were run using the GTR-Γ model. RAxML was run with 8 threads specifically, and FastTree was run without specifying the number of threads. The commands are:

RAxML

```
raxmlHPC-PTHREADS-AVX -s <alignment_fasta> -w <work_folder> -n <tree_name>.tre
-m GTRGAMMA -p 100 -T 8
```

FastTree-2

```
FastTreeMP -quiet -gtr -gamma -nt <alignment_fasta> > <tree_name>.tre
```

3 Additional Scatter Plots for 1000-sequence Data

In this section, we present all three pairwise comparisons of all metrics on the 1000-sequence data. The three methods considered are, briefly:

a. **PASTA(Default)** PASTA under default settings: 3 iterations, all with MAFFT (L-Ins-I) as the subset aligner and decomposition to a maximum size of 200 sequences.

   For each of the next two methods, we run PASTA for 1 iteration with the initial tree taken from the output of this method, which is equivalent to running PASTA for 4 iterations with slightly different settings on the final cycle.

b. **PASTA+BAli-Phy** PASTA for 1 iteration, with the BAli-Phy Posterior Decoding alignment on subsets and decomposition to a maximum size of 100 sequences. Using the PASTA(default) output phylogeny as an initial tree.

c. **PASTA+MAFFT-L** PASTA for 1 iteration, with MAFFT L-Ins-I as the subset aligner and decomposition to a maximum size of 100 sequences. Using the PASTA(default) output phylogeny as an initial tree.

Figure 1 shows full results (for all five criteria) corresponding to Figure 1 from the main paper, which is partially duplicated here. In all subfigures in this section, each point represents one replicate and a position above or below the 45-degree line is interpreted as favoring one method or the other consistently across the page. This requires inverting the axes for the bottom panels compared to the upper three, but maintains a consistent interpretation.

Note that the figures for SP-score and Modeller score are nearly identical. Delta-FN results using FastTree-2 and RAxML have some differences in terms of magnitude, but not in terms of relative performance. The remaining figures are the equivalents of Figure 1 in the discussion section of the paper, with the difference that each compares a different pair of methods. The second compares PASTA+MAFFT-L to default PASTA . The fourth iteration with MAFFT L-INS-i improves the TC score but does not improve either precision or recall. The comparison with respect to precision and recall shows that PASTA+MAFFT-L is better than default PASTA on the Indelible datasets, but about the same on the RoseDNA data, and less accurate than default PASTA on the RNAsim data.

The final figure compares PASTA+BAli-Phy to PASTA+MAFFT-L directly. Consistent with the previous figure, PASTA+BAli-Phy seems to be much better than PASTA+MAFFT-L in much the way that it was better on PASTA run in default setting.

4 Comparison of RAxML vs. FastTree-2

Table 1 gives a detailed comparison of the actual error rates for each of the two maximum-likelihood tree estimation techniques.

Table 1: **RAxML and FastTree-2 RF error details** for each dataset and each method. On the Indelible data, FastTree-2 generates a tree that is noticeably less accurate than RAxML for all alignments, including the refrence, and it is not clear what causes that. On all other data the two perform about equally.

		RF Error		
**Data**	**Alignment**	**RAxML**	**FT-2**	**Diff.**
**Indelible M2**	P(default)	24.1%	32.2%	-8.1%
	P(BAli-Phy)	22.6%	30.8%	-8.2%
	P(MAFFT)	23.0%	31.3%	-8.3%
	MAFFT	0.0%	0.0%	0.0%
	Reference	22.3%	31.5%	-9.2%
**RNAsim**	P(default)	16.1%	16.4%	-0.3%
	P(BAli-Phy)	16.3%	16.5%	-0.3%
	P(MAFFT)	15.9%	16.5%	-0.6%
	MAFFT	0.0%	0.0%	0.0%
	Reference	15.6%	16.1%	-0.5%
**Rose L1**	P(default)	14.0%	13.4%	0.6%
	P(BAli-Phy)	13.4%	12.7%	0.7%
	P(MAFFT)	13.9%	13.2%	0.7%
	MAFFT	0.0%	0.0%	0.0%
	Reference	11.9%	11.2%	0.7%
**Rose M1**	P(default)	16.8%	17.0%	-0.1%
	P(BAli-Phy)	16.2%	16.1%	0.1%
	P(MAFFT)	17.5%	16.6%	0.9%
	MAFFT	0.0%	0.0%	0.0%
	Reference	11.5%	10.7%	0.8%
**Rose S1**	P(default)	14.7%	14.0%	0.7%
	P(BAli-Phy)	13.0%	13.3%	-0.2%
	P(MAFFT)	14.3%	14.1%	0.3%
	MAFFT	0.0%	0.0%	0.0%
	Reference	10.8%	9.7%	1.1%

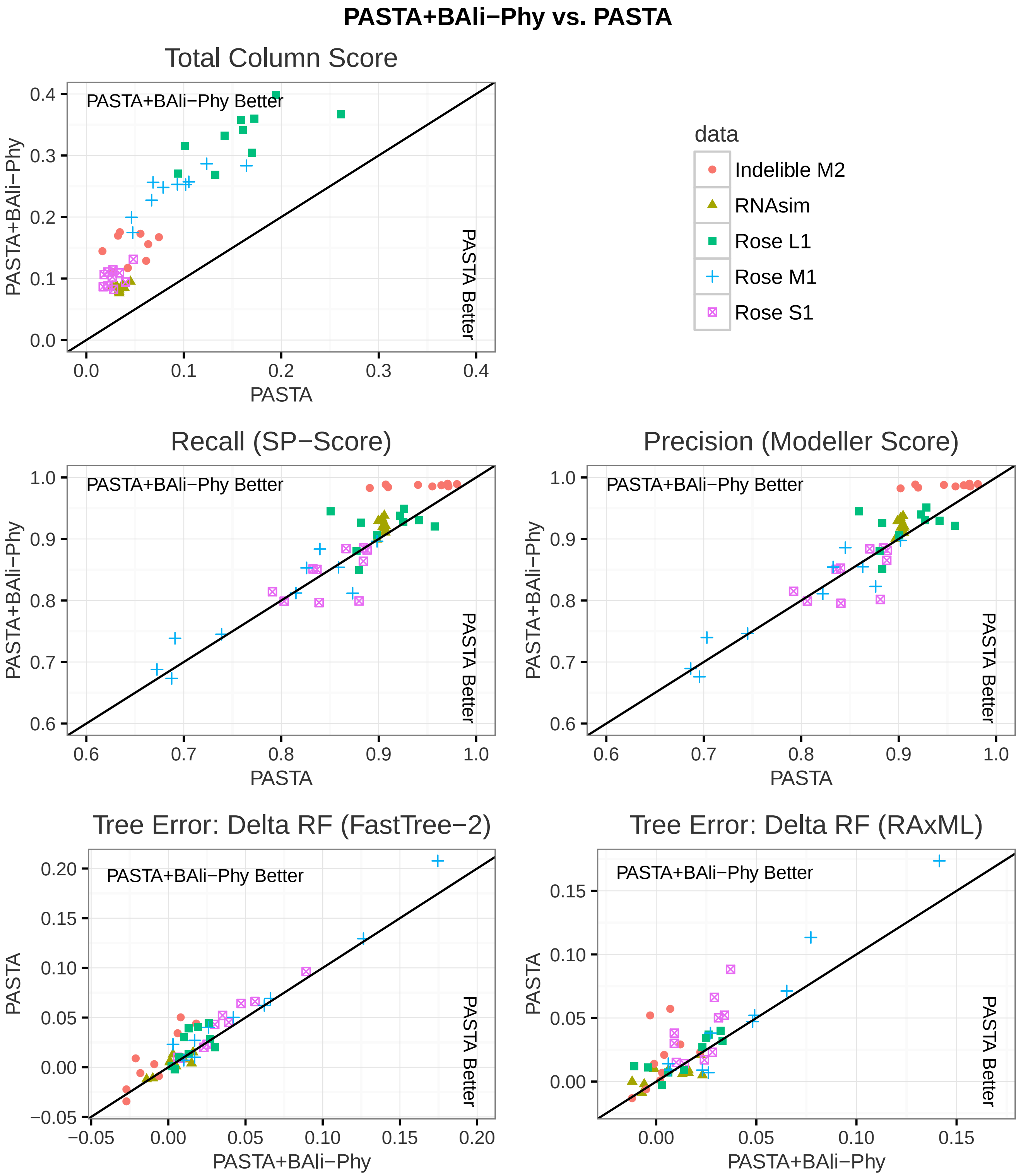

Figure 1: Pointwise comparison of five criteria for PASTA before and after a final iteration using BAli-Phy as the subset aligner. PASTA denotes the alignment from PASTA under default settings (referred to as "PASTA(default)" in the text). Delta-RF refers to the difference between the RF error rates of ML trees computed on the estimated and true alignments.

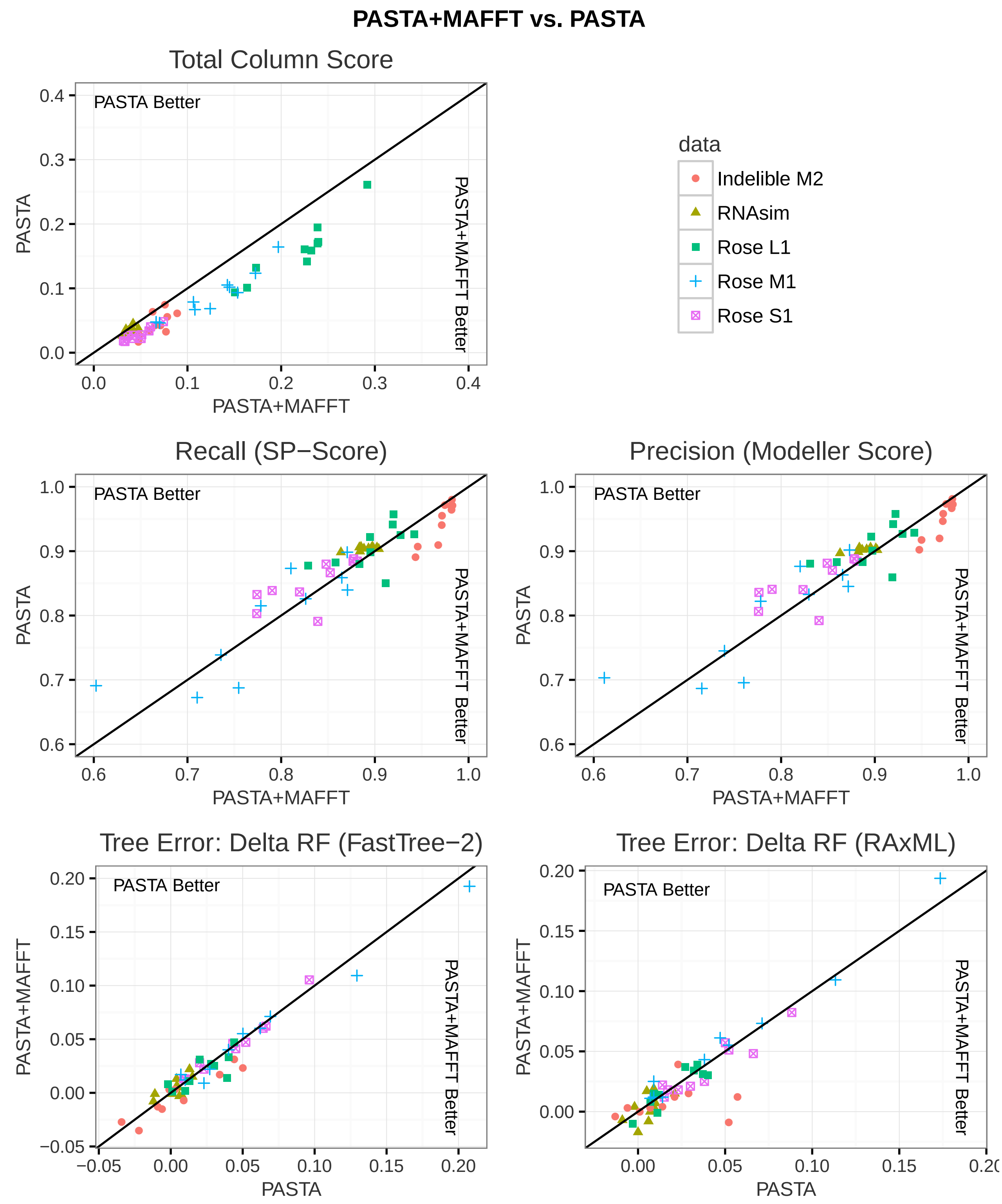

Figure 2: Pointwise comparisons between PASTA run in default setting (referred to as PASTA here) and PASTA+MAFFT-L, analogous to Figure 1. It appears that the fourth iteration with MAFFT improves the TC score but does not improve either precision or recall. PASTA+MAFFT-L is better for precision and recall on Indelible, but worse on RNAsim and roughly even on RoseDNA.

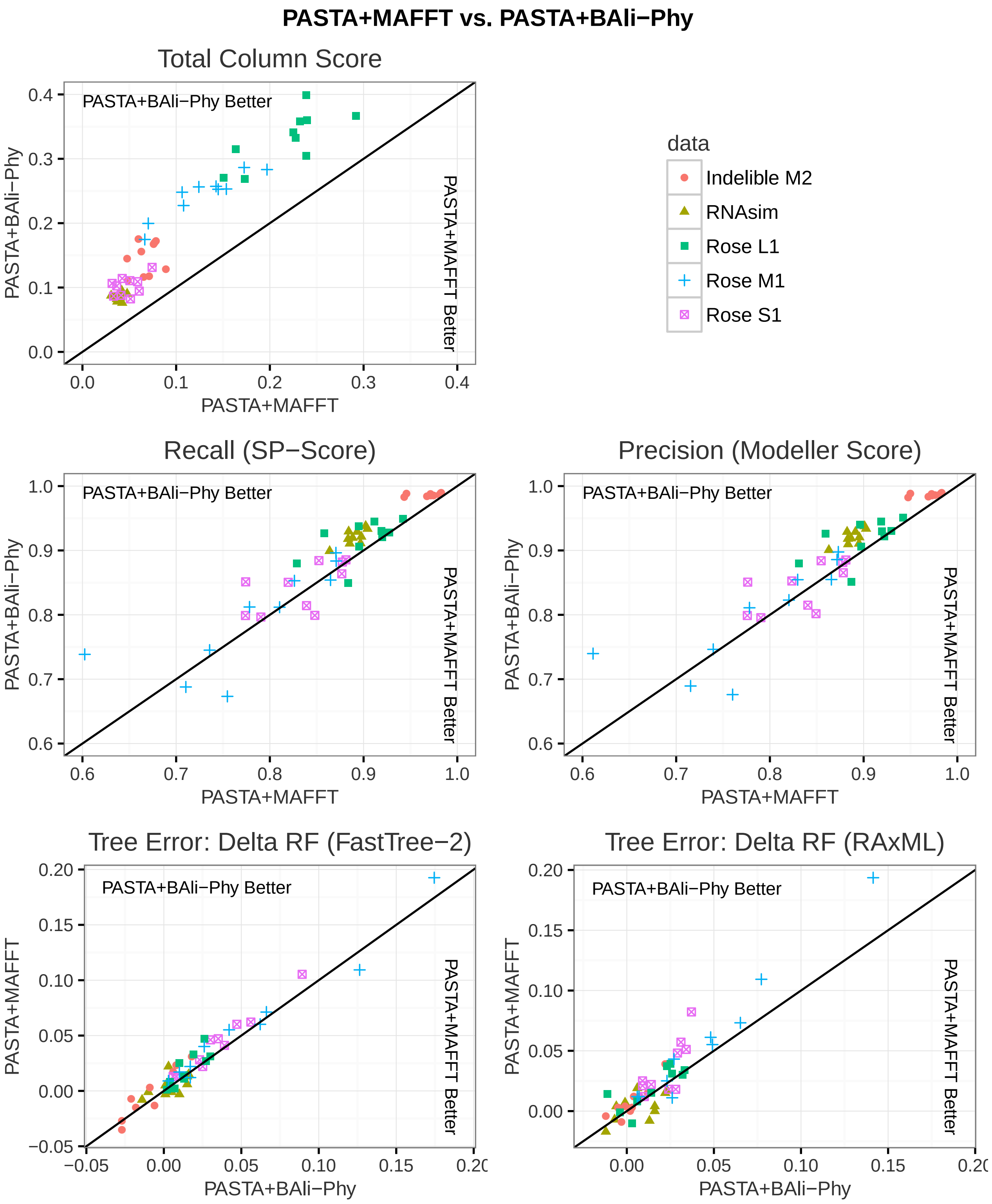

Figure 3: Same as Figure 2, but comparing PASTA+MAFFT-L to PASTA+BAli-Phy. Recall that PASTA+BAli-Phy refers to a single iteration of PASTA run with BAli-Phy as the subset aligner on subset-size 100, using the tree from PASTA as the starting tree. PASTA+MAFFT-L is analogous with MAFFT in place of BAli-Phy.
